# Oleic Acid and Succinic Acid Synergistically Mitigate Symptoms of Type 2 Diabetes in Streptozotocin-Induced Diabetic Rats

**DOI:** 10.1155/2022/8744964

**Published:** 2022-02-27

**Authors:** K. G. Lattibeaudiere, R. L. Alexander-Lindo

**Affiliations:** Department of Basic Medical Sciences, Biochemistry Section, The University of the West Indies, Mona, Kingston, Jamaica

## Abstract

Succinic acid (SA) and oleic acid (OA) are the primary hypoglycaemic agents in *Desmodium canum,* a plant traditionally employed for its potential health benefits. The synergy of the two organic acids exhibits potency in retarding blood glucose levels (BGL) in euglycaemic Sprague Dawley (S-D) rats following a single-dose administration. A cocktail of the two compounds is being investigated for its antidiabetic properties in fructose-fed streptozotocin (STZ)-induced diabetic rats. Eighteen type 2 diabetic S-D rats were divided into 3 groups and treated for 28 d with either the cocktail (OA + SA, 800 mg/kg body weight [BW]), glibenclamide (10 mg/kg BW), or vehicle (10% polysorbate 20). Another 12 S-D rats served as euglycaemic animals and were divided into two groups, fed either the cocktail (OA + SA, 800 mg/kg BW) or vehicle. Changes in BW, blood pressure (BP), BGL, water and food consumption, serum insulin, serum glucagon and insulin resistance (IR) were monitored. Treatment with the cocktail showed no change in euglycaemic animals; however, there was a significant reduction in the BGL of diabetic treated animals when compared with diabetic control (14.48 ± 1.92 vs. 25.56 ± 1.38 mM; *p*=0.012). Quantitative insulin sensitivity check index (QUICKI) and glucose/insulin (G/I) scores for IR indicated an improvement in insulin response in the diabetic treated animals. Additionally, there was a noticeable reduction in food and water consumption when compared with diabetic control animals, which was accompanied by a reduction in weight. Overall, the cocktail elicited antidiabetic properties and may serve an important therapeutic role as a nutritional supplement in type 2 diabetics.

## 1. Introduction

Diabetes mellitus (DM) continues to be a challenging issue that plagues many Caribbean territories. In fact, it accounts for as much as 13.8% of all deaths among adults within the region [1]. As defined by the American Diabetes Association(ADA), the condition is a group of metabolic diseases that are characterized by chronic hyperglycaemiadue to a deficiency in insulin secretion, insulin's action, or both [2]. Hyperglycaemia, as a result of diabetes, disrupts the physiological homeostasis and exacerbates the condition through a wide array of complications. These may initially be mild conditions such as ease of exhaustion, polyuria, polyphagia, and polydipsia [3–5]. However, as a progressive disease, there may be the development of cardiovascular complications, endothelial dysfunction, neuropathy, nephropathy, nontraumatic amputations, among others [4, 6]. Several of these are as a result of the development of oxidative stress which is well documented to be strongly correlated with DM [7]. This includes the noticeable positive correlation between diabetes and hypertension, with as much as two times more diabetics being diagnosed with the disease. Several researchers have postulated that this may be due to several factors including an increase in advanced glycated end products (AGEs), dyslipidemia, and oxidative stress [8]. These contribute to the reduction of the elasticity of the arteries or reduce blood flow, hence elevating the pressure of blood being exerted on the walls of the arteries.

Owing to its severity, DM is often accompanied by financial constraints in developing countries. Annual treatment for diabetic patients costs the Jamaican government millions of US dollars, with this trend likely to increase [9]. Many locals rely on folklore medicine as their sole source of treatment [10–12]. This essentially includes the use of plants, animals, and/or minerals to alleviate the effects of the disease [13, 14]. Many scientific researches have added credence to this source of therapy by highlighting the effects of traditional medicine and have elucidated the bioactive components [15–17]. For example, the common plant, *Desmodium canum*, is consumed locally for its belief to be an aphrodisiac, lower BGL, and reduce the effect of asthma [11]. Recent studies have demonstrated that the hexane extract increases serum testosterone levels in male S-D rats [17] and the ethyl acetate extract possesses a hypoglycaemic effect in normal S-D rats. Lattibeaudiere and colleagues [18] further demonstrated that the hypoglycaemic activity observed in the normal rats was due to the synergistic action of OA and SA found in the plant. Both compounds have been documented to individually possess a hypoglycaemic activity, but as a cocktail, the potency rivals that of metformin in euglycaemic S-D rats [18].

SA exists as a dicarboxylic acid and plays a central role in the metabolism of glucose through the tricarboxylic acid (TCA) cycle. When administered exogenously, SA reduces BGL through insulin secretion [19]. De Marchi et al. highlighted the role of SA in insulin secretion even in low glucose levels [20]. The monounsaturated fatty acid, OA has also been shown to improve glycaemic control and to improve insulin sensitivity. However, to the best of our knowledge, the synergistic effect of these compounds in type 2 diabetic rats has never been investigated. We, therefore, aim to investigate the effect of these compounds as a nutritional supplement in fructose-fed STZ-induced type 2 diabetic rats.

## 2. Method

### 2.1. Ethical Considerations

Ethical approval was granted for the use of animals by the Faculty of Medical Sciences, UWI, Mona Campus Ethics Committee.

### 2.2. Materials and Reagents

STZ, SA (bioXTra, >99%), OA (bioreagent, >99%), polysorbate 20, glibenclamide (5 mg/kg BW was used in the antidiabetic drug response test and 10 mg/kg BW was used for the daily treatment of a group of diabetic rats), and fructose were purchased from Sigma Aldrich. Insulin rat ultrasensitive ELISA kit and glucagon rat ELISA kit were purchased from Crystal Chem Inc., USA; Formulab diet 5008 (crude protein >23%, crude fat >6.5%, crude fiber >4.0%, ash >8.0%, and moisture >12.0%) purchased from LabDiet, USA; CODA-6 Noninvasive Blood Pressure System (Kent Scientific).

### 2.3. Diabetic Induction and Treatment

Thirty S-D rats (15 males and 15 females) of a mean weight of 155 g were obtained from the UWI Basic Medical Sciences animal house and divided into 5 main groups (*n* = 6). These groups were further subdivided to accommodate 3 rats per cage based on their gender. The animals were kept at room temperature and exposed to 12 h light/dark cycles. The experiment protocol was adopted from Wilson and Islam with modifications [21]. The rats were fed Formulab diet 5008 and either tap water (groups 1 and 2) or 10% fructose solution (groups 3–5) ad libitum for 14 days. At the end of this period, the rats were deprived of food for approximately 12 h and the diabetic groups were administered STZ intraperitoneally (40 mg/kg BW) dissolved in citrate buffer (0.3 ml, pH 4.5) while the normal groups were injected with a similar volume of the citrate buffer. One week after the STZ administration, the nonfasting BGL greater than 15.5 mmol/L were considered to be diabetic. The groups were treated for 4 weeks with either 10% polysorbate 20, glibenclamide, or a combination of OA and SA. The dose of each was chosen based on previous studies and is outlined as follows [18, 22]:

  Group 1: normal control (NC): daily administration of 0.5 mL of 10% polysorbate 20
  Group 2: normal treated (NT): daily administration of OA + SA (1 : 1, 800 mg/kg BW)) dissolved in 0.5 mL of 10% polysorbate 20
  Group 3: diabetic control (DC): daily administration of 0.5 mL of 10% polysorbate 20
  Group 4: diabetic glibenclamide (DGlib): daily administration of glibenclamide (10 mg/kg BW) dissolved in 0.5 mL of 10% polysorbate 20
  Group 5: diabetic treatment (DT): daily administration of OA + SA (1 : 1, 800 mg/kg BW) dissolved in 0.5 mL of 10% polysorbate 20

Food and water intake were measured daily while BGL, BW, and BP were measured on a weekly basis. After 28 days of treatment, the animals were deprived of food overnight and then sacrificed via sodium pentobarbital injection (intraperitoneally, 65 mg/kg BW).

### 2.4. Antidiabetic Drug Response Test

The antidiabetic drug response test was used to determine the type of model induced. Two days after the confirmation of diabetes, the BGL of 3 h fasted rats were measured using an Accu-Chek glucometer. A low dose of glibenclamide (5 mg/kg BW) was administered by oral gavage and the animals returned to their cages. After a further 3 h, BGL were monitored once more and statistical comparisons were made between the two readings. If there was a significant decrease in BGL following treatment with glibenclamide, this acts as an indicator of a type 2 model.

### 2.5. Blood Collection and Treatment

Blood (6 mL) was removed from the renal arteries of the animals and transferred to a red top vacutainer. These were then centrifuged at 2200 rpm for 15 min. Sera were obtained and analysed for insulin and glucagon levels using rat ultrasensitive insulin ELISA kit and rat glucagon ELISA kit, respectively, purchased from CrystalChem Inc.

### 2.6. Measuring Insulin Sensitivity

Insulin sensitivity was assessed using QUICKI and G/I shown below [23]:

QUICKI:
  (1) $$\begin{array}{c} \frac{1}{\left(\log\text{ fasting glucose}\right)+\log\text{ fasting insulin}}. \end{array}$$
G/I ratio:
  (2) $$\begin{array}{c} \frac{\text{fasting glucose}\left(\text{mg}/\text{dL}\right)}{\text{fasting insulin levels}\left(\text{mg}/\text{dL}\right)}. \end{array}$$

### 2.7. Blood Pressure Measurement

The systolic blood pressure (SBP), diastolic blood pressure (DBP), and mean arterial pressure (MAP) of the animals were measured on a weekly basis from week 1 to week 6 using the CODA 6 noninvasive pressure machine (Kent Scientific). Normal BP was considered at SBP∼128 mmHg, DBP∼95 mmHg, and MAP∼106 mmHg [17]. During week 0, animals were acclimatized to the machine and the experiment was done similar to the study carried out by Logan and colleagues [24]. This was done by placing the rats in a restraining chamber for four separate 15 min periods for three days. On experimental days, the animals were placed in the chamber and the cuffs (occlusion and the volume pressure recorder sensor) were placed on the tails of the animals. The machine cycles were run and BP for each rat was monitored within 10 min. These were averaged to reflect one reading per rat.

### 2.8. Statistical Analysis

Data were expressed as mean ± standard error of the mean, while comparisons among the groups were assessed using one-way ANOVA followed by Tukey post hoc test (IBM SPSS Statistics for Windows, version 20 [IBM Corp., Armonk, NY, USA]), where *p* ≤ 0.05 was considered to be statistically different.

The influence of gender on both the BGL and BP was assessed using Spearman's correlation test.

## 3. Results

### 3.1. Antidiabetic Drug Response Test

**Figure 1:**
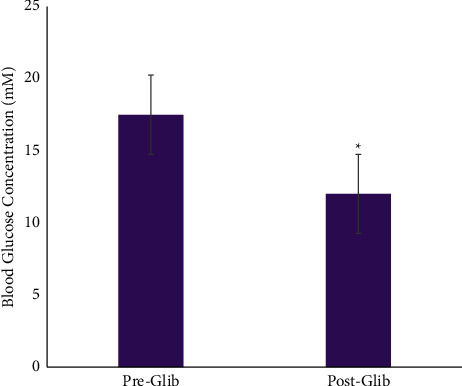
Antidiabetic drug response test of diabetic animals used in the study, where ^*∗*^indicates a statistically significant difference when compared with preglibenclamide administration.

In the antidiabetic drug response test, a low dose of glibenclamide (5 mg/kg) was administered. This significantly reduced the BGL of the diabetic rats as seen in Figure 1 (*p*=0.029). The difference between the preglibenclamide and postglibenclamide administration indicates a positive response, suggesting the presence of functional *β*-cells of the pancreas.

### 3.2. The Weekly Change in Blood Glucose Levels of Diabetic and Nondiabetic Sprague Dawley Rats

The cocktail supplement of OA + SA resulted in a gradual reduction in the BGLs of diabetic animals, which was significantly lower than DC at weeks 6 (14.82 ± 2.31 mM vs. 28.53 ± 2.75 mM; *p*=0.04) and 7 (14.48 ± 1.92 mM vs. 25.56 ± 1.38 mM; *p*=0.012). However, the cocktail failed to reduce the BGL of these animals to normal levels (NC and NT groups). Similarly, the glibenclamide treated animals had a similar response as the DT group with a gradual reduction in BGL until weeks 6 and 7 where it became significantly lower than the DC animals (*p*=0.04 and *p*=0.012, respectively). There were no statistically significant differences between DT and DGlib. Additionally, the cocktail had no significant effect on the BGL of the euglycaemic animals (*p* > 0.05).

### 3.3. Food and Water Consumption

**Figure 2:**
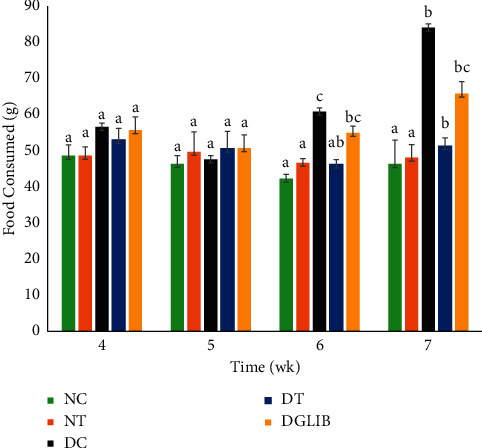
Average daily food consumption of diabetic and euglycaemic S-D rats in the last four weeks of the study. Data are expressed as mean ± standard error of the mean and analysed by tukey post hoc statistical test. NC: normal control, NT: normal treatment, DC: diabetic control, DT: diabetic treatment, and DGlib: diabetic glibenclamide group, where the letters represent the following: a, statistically significant differences when compared with the bars with letters b and c; b, statistically significant differences when compared with the bars with letters a and c; c, statistically significant differences when compared with the bars with letters a and b; ab, no significant difference when compared with the bars with either a or b; bc, no significant difference when compared with the bars with either b or c. Significant difference is considered at *p* ≤ 0.05.

**Figure 3:**
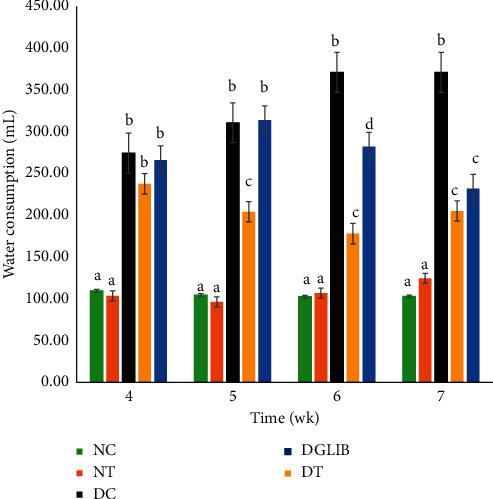
Average daily water intake of diabetic and euglycaemic Sprague Dawley rats in the last four weeks of the study. Data are expressed as mean ± standard error of the mean and analysed by Tukey post hoc statistical test. NC: normal control, NT: normal treatment, DC: diabetic control, DT: diabetic treatment, and DGlib: diabetic glibenclamide group, where the letters represent the following: a, statistically significant differences when compared with the bars with letters b, c, and d; b, statistically significant differences when compared with the bars with letters a, c, and d; c, statistically significant differences when compared with the bars with letters a, b, and d; d, statistically significant differences when compared with the bars with letters a and b. Significant difference is considered at *p* ≤ 0.05.

The manifestation of polyphagia and polydipsia is common symptoms of diabetes in humans and is expressed in the model used in the study. The DC animals showed a marked increase in food consumption over the latter 4 weeks of the study (Figure 2), with week 7 showing an advanced stage of polyphagia as compared to NC (*p* < 0.001). The synergy of OA and SA regulated food intake of diabetic animals, where food consumption was significantly reduced by week 7 when compared with DC (*p* < 0.05), though still higher than the euglycaemic animals.  Figure 3 depicts the exhibition of polydipsia in the diabetic animals, where the cocktail ameliorated the symptom by reducing the volume of water consumed when compared with DC (week 7; *p* < 0.001). The glibenclamide treated animals showed a similar response to that of the DT animals. On the other hand, the cocktail supplement showed no change in food or water intake of the euglycaemic animals when compared with NC (*p* > 0.05).

### 3.4. Serum Insulin, Glucagon, and Insulin Resistance

Table 1 highlights the variation in serum insulin, glucagon, and IR of the animals used in the study. There were no statistically significant differences in the serum insulin levels among the groups (*p* > 0.05). On the other hand, the cocktail treatment significantly reduced the serum glucagon levels when compared with the DC animals (*p*=0.04). The levels of glucagon were reduced to values that are comparable to that of NC (*p* > 0.05). Simultaneously, as compared with the DC, the cocktail improved IR as seen in both the QUICKI (*p*=0.02) and G/I ratio (*p*=0.026). Glibenclamide had no effect on the glucagon levels when compared to both the NC and DC groups (*p* > 0.05) but showed an improvement in IR with both QUICKI (*p*=0.03) and G/I ratio (*p*=0.006). There were no changes for any of the parameters for the NT group when compared with the NC group (*p* > 0.05).

### 3.5. The Effect on Body Weight of the Animals

**Figure 4:**
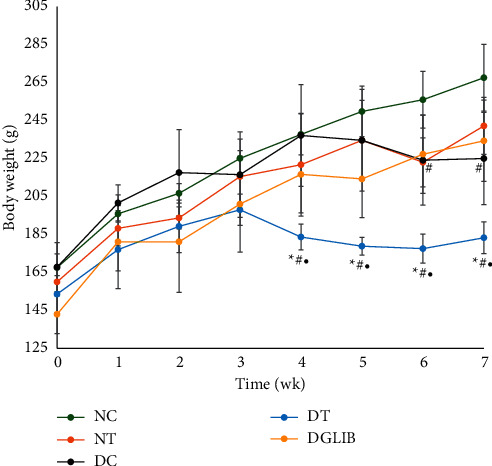
The effect of the cocktail on the body weight of the animals over the period of the study. Data are expressed as mean ± standard error of the mean and analysed by Tukey post hoc statistical test. NC: normal control, NT: normal treatment, DC: diabetic control, DT: diabetic treatment, and DGlib: diabetic glibenclamide group, where ^#^indicates *p* ≤ 0.05 when compared with NC. ^*∗*^indicates *p* ≤ 0.05 when compared with DGLIB. ^•^indicates *p* ≤ 0.05 when compared with DC; significant difference is considered at *p* ≤ 0.05.

The synergy of OA and SA resulted in a significant reduction of the BW of diabetic animals when compared with all the other groups (*p* < 0.05). This reduction was seen as early as week 4 and continued up until the end of the experiment in week 7. DC also showed a significant reduction in BW when compared with NC. This was only observed at the end of the experiment in weeks 6 and 7 in Figure 4. None of the other groups showed any statistically significant changes in the BW of the animals.

### 3.6. The Effect on Blood Pressure

**Figure 5:**
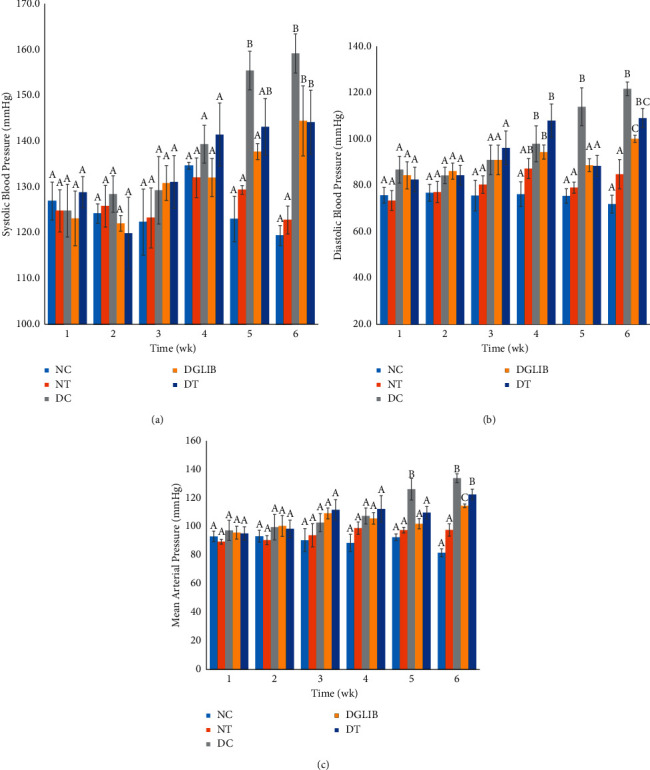
(a) The effect of the cocktail on the SBP of diabetic and euglycaemic S-D rats. Data are expressed as mean ± standard error of the mean and analysed by Tukey post hoc statistical test. NC: normal control, NT: normal treatment, DC: diabetic control, DT: diabetic treatment, and DGlib: diabetic glibenclamide group, where the letters represent the following: a, statistically significant differences when compared with the bars with letters b and c; b, statistically significant differences when compared with the bars with letters a and c; c, statistically significant differences when compared with the bars with letters a and b; ab, no significant difference when compared with the bars with either a or b. Significant difference is considered at *p* ≤ 0.05. (b) the effect of the nutritional supplement on the DBP of diabetic and euglycaemic S-D rats. Data are expressed as mean ± standard error of the mean and analysed by Tukey post hoc statistical test. NC: normal control, NT: normal treatment, DC: diabetic control, DT: diabetic treatment, DGlib: diabetic glibenclamide group, where the letters represent the following: a, statistically significant differences when compared to the bars with letters b and c; b, statistically significant differences when compared with the bars with letters a and c; c, statistically significant differences when compared with the bars with letters a and b; ab, no significant difference when compared with the bars with either a or b; bc, no significant difference when compared with the bars with either b or c. Significant difference is considered at *p* ≤ 0.05. (c) The effect of the nutritional supplement on the MAP of diabetic and euglycaemic S-D rats. Data are expressed as mean ± standard error of the mean and analysed by Tukey post hoc statistical test. NC: normal control, NT: normal treatment, DC: diabetic control, DT: diabetic treatment, and DGlib: diabetic glibenclamide group, where the letters represent the following: a, statistically significant differences when compared to the bars with letters b and c; b, statistically significant differences when compared to the bars with letters a and c; c, statistically significant differences when compared to the bars with letters a and b. Significant difference is considered at *p* ≤ 0.05.

Figures 5(a)–5(c) highlight the changes in the BP of the animals used in the study. In Figure 5(a), the elevation of the SBP of diabetic animals is seen, with DC showing the highest levels. Between weeks 1 and 4, there were no statistically significant differences in the SBP among the groups. However, week 5 shows a significant elevation in the SBP of the DC animals when compared with the NC group (*p* < 0.001). DT animals showed no elevation in their SBP until week 6, where it was significantly higher than NC (*p*=0.010) and showed no difference when compared with DC (*p* > 0.05). The DBP seen in Figure 5(b) showed an earlier onset of elevation, where the DC group showed an elevation in the parameter by week 4 (*p*=0.016) and progressively increase up to 121.70 ± 2.94 mmHg by week 6. The cocktail showed the most significant reduction in the DBP when compared with the DC at week 5 (*p*=0.015). However, this was short-lived as the DBP elevated by week 6 with no statistical difference when compared with DC and NC. On the other hand, glibenclamide had a positive effect on reducing the DBP of diabetic rats in week 5 (*p*=0.017) and week 6 (*p*=0.02). A similar pattern was seen in Figure 5(c) where the MAP of the DC group was significantly greater than NC at week 5 (*p* < 0.001). The cocktail delayed the elevation until week 6, where there was no significant difference in the MAP between DC and DT (*p* > 0.05). Glibenclamide, however, showed potency in reducing the MAP when compared with the DC animals (*p*=0.005).

## 4. Discussion

The present study highlights the antidiabetic effect of a nutritional supplement of a cocktail of OA and SA. This was achieved through the successful induction of type 2 diabetes in a rat model using 10% fructose and low dose STZ (40 mg/kg BW). Fructose has been noted to upregulate the synthesis of long-chain fatty acids which are esterified to diacylglycerides [25, 26]. Elevated levels of serum lipids in both humans and animal models have been documented to increase IR which is consistent with the characteristics of type 2 diabetes. Generally, IR precedes the development of the metabolic condition, DM, which was mimicked in this study. Administration of STZ further exacerbated the condition through damage of the *β*-cells of the pancreas. Figure 1 depicts the effect of the insulinotropic agent, glibenclamide, on BGL in the diabetic animals. In this, the BGL of the rats were reduced from 17.50 ± 0.78 mM to 12.01 ± 1.06 mM (*p* < 0.001), indicating a positive response to the drug. Studies have reported that glibenclamide has no effect on BGL in type 1 diabetic model [27]; hence, a significant reduction of BGLs in the diabetic rats is usually an indicator of a type 2 model. This is as a result of the pharmacodynamics of the drug in promoting insulin release; however, type 1 models generally produce little to no insulin and as such, glibenclamide fails to reduce the BGL of these animals.

Furthermore, IR was achieved as shown in Table 1, represented by QUICKI and G/I ratio. Several studies have added credence to the usefulness of these methods in quantifying insulin resistance [22]. Notably, as mentioned, IR is generally associated with type 2 diabetes which further exacerbates the condition through reduce uptake of glucose into the peripheral tissues such as the skeletal muscles and the liver. The use of the cocktail successfully attenuated IR indiabetic animals. Using QUICKI, it has been noted that there was a successful restoration of insulin sensitivity due to treatment with OA + SA. The cocktail brought IR values to those comparable with the NC group (*p*=1.00). Moreover, there was a significant reduction in IR when compared with the DC (*p*=0.047). Similarly, this was seen in the G/I ratio, where the cocktail significantly reduced the ratio, indicating successful amelioration of insulin sensitivity in the diabetic rats fed OA + SA. The presence of the monounsaturated fatty acid, OA, is responsible for the removal of deleterious materials from peripheral tissues that may promote IR. Additionally, the improvement in insulin sensitivity also alleviated the insulin-induced suppression of glucagon release from the *α*-cells of the pancreas. Elevation of glucagon secretion is related to the aberrant BGL reported in DC, as the hepatic output of glucose is increased. However, with the alleviation in IR and reduction in glucagon levels in the DT group, there is an improvement in glucose tolerance in these diabetic animals. Glibenclamide treatment animals also showed a similar response to their treatment which was reflected in an improvement of insulin sensitivity with values of 0.334 ± 0.002 and 1.951 ± 0.263 × 10^3^ using QUICKI and G/I ratio, respectively (Table 1).

Interestingly, there were no differences in the fasting serum insulin levels among the groups. It has been reported in the literature that treatment with glibenclamide has no effect on fasting insulin levels due to the mechanism of action. The insulinotropic property of glibenclamide stems from the glucose responsiveness of the pancreatic *β*-cells [27, 28]. Consequently, the sulfonylurea drug is most potent in the fed state rather than the fasting state, hence no change in the fasting serum insulin as outlined in Table 1. Moreover, the lack of evidence of a significant difference between DC and the euglycaemic groups (NC and NT) may be as a result of the status of the diabetic model. Foster et al. corroborated this finding, where they reported that there is no difference in the fasting serum insulin levels between type 2 diabetic and nondiabetic models used in their study [29]. Similarly, treatment with the cocktail showed no response in the fasting insulin levels in neither diabetic nor euglycaemic groups. OA has previously been reported to attenuate the symptoms of diabetes via increasing insulin secretion and decreasing the deleterious products that facilitate IR [30]. SA has also been reported to stimulate the release of insulin [19]. In a previous study, we reported that the cocktail failed to reduce BGL in the fasting state of euglycaemic rats, but there was a significant decrease in the postprandial state [18]. This corroborates the argument being made that the cocktail may be most potent in promoting insulin secretion in the fed state.

**Figure 6:**
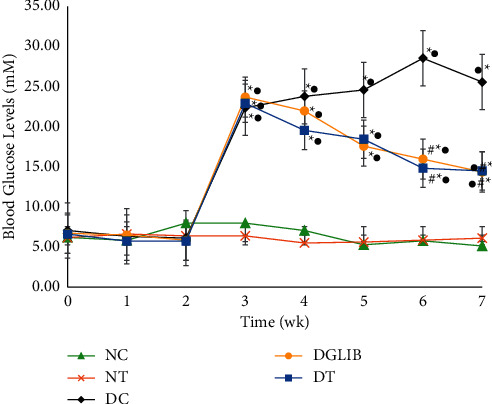
The change in BGL of diabetic and euglycaemic S-D rats following treatment for 28 d. Data are expressed as mean ± standard error of the mean and analysed by Tukey post host statistical test. NC: normal control, NT: normal treatment, DC: diabetic control, DT: diabetic treatment, DGlib:diabetic glibenclamide group. ^*∗*^indicates statistically significant difference when compared with NC. ^#^indicates statistically significant difference when compared with DC. ^•^indicates statistically significant difference when compared NT, where statistically significant difference is expressed as *p* ≤ 0.05.

Despite no change in the fasting serum insulin, there was a noticeable decrease in the BGL of diabetic animals treated with the cocktail.  Figure 6 highlights the progressive improvement of glucose metabolism in the animals used throughout the study. The cocktail significantly reduced the aberrant glucose levels seen at week 3, through a synergy of improved insulin sensitivity and the insulinotropic nature of SA. This was most noticeable in the last two weeks of the study where there was a significant difference between DC and DT (28.53 ± 2.75 vs. 14.82 ± 2.31 mM; *p*=0.04) and continued into week 7 (25.56 ± 1.38 vs. 14.48 ± 1.92 mM; *p*=0.012). The reduction of BGL in the diabetic treatment group showed no significant difference when compared with glibenclamide treated animals, suggesting a similar potency. The reduction stems from the improvement of IR, which means insulin is better able to promote uptake of glucose into the peripheral tissues, as well as a reduction in the glucose output.

On the other hand, there was no change in the BGL of the euglycaemic animals treated with the cocktail. Tomita and colleagues reported that OA only causes glucose-stimulated insulin release when there is a certain level of glucose present within the blood [31]. No study has reported the effect of SA on BGL in euglycaemic patients, but it is possible that the insulinotropic nature of the dicarboxylic acid may be glucose-dependent. Consequently, the synergy of OA and SA failed to alter the BGL of euglycaemic animals, which may suggest that the cocktail can be consumed without the risk of a hypoglycaemic shock. Furthermore, as described above, in a separate study, it was noted that the cocktail only reduced the BGL of euglycaemic rats only after glucose was administered orally [18]. This further suggests that there is a threshold of glucose needed within the blood for the cocktail to cause lowering; thus, it is highly unlikely that consumption will promote a hypoglycaemic shock.

Many studies opt to use male rats in diabetic research for fear of hormonal factors being an influence in glucose metabolism in female rats. However, it is being highlighted that gender played no role in the glucose levels in the research. This was inferred from Spearman's correlation test that suggested gender played no role in the BGL in both diabetic and nondiabetic rats (*r*_*s*_ = 0.12, *p*=0.535).

The retardation of BGL in the diabetic animals was accompanied by alleviating symptoms associated with diabetes such as polyphagia and polydipsia as seen in Figures 2 and 3, respectively. These symptoms are typically associated with hyperglycaemia, thus alleviating this condition tends to reduce polyphagia and polydipsia. This further indicates the efficacy of the antidiabetic properties of the cocktail and may potentially offer significant health benefits in treating type 2 diabetics. However, there was a significant reduction in the weight of the animals fed the cocktail. Interestingly, these animals showed the greatest retardation in growth and were significantly lower than all the other groups. Liu and colleagues reported that diets rich in OA show a significant reduction in central obesity [32]. This has been corroborated by several other studies which highlight a reduction in weight following diets rich in OA [33, 34]. Moreover, regulation of food intake by the cocktail in weeks 6 and 7 may partially be responsible for the reduction in the BW of the animals. The cocktail being rich in OA also played a role in the reduction of the weight of diabetic animals; however, this was not observed in the nondiabetic animals. The possibility of the cocktail being used to treat obese diabetics may therefore be explored with further studies.

The treatment using the cocktail failed to reduce the BP of diabetic animals. Several studies have documented the correlation between diabetes and hypertension [4, 35, 36]. In these, diabetes linked to hypertension is usually due to an increase in oxidative stress, glycation of the smooth muscles of the arteries, and dyslipidemia [8], all of which are elevated through disruption of homeostasis stemming from hyperglycaemia. As with an increase in BGL, the BP of diabetic animals progressed throughout the study. This was evident in the DC group which showed a 23.8% increase in SBP and a 40.2% increase in DBP between week 1 and week 6. Though there was only a mild significant difference between the DT and DC groups in these blood pressure parameters (week 5), there was only an 11.9% increase in the SBP and a 32.14% increase in DBP. Overall, this increase was lower than that of the diabetic untreated group, perhaps indicating a potential to reduce the risk of severe hypertension in diabetics. The glibenclamide treated group showed a more significant decrease in blood pressure with a 17.4% increase in SBP and a 21.0% increase in DBP. This may be as a result of the mechanism of action of the sulfonylurea drug. The reduction in BGL over time may result in a reduction in oxidative attack or glycation of the arteries and may therefore improve the blood pressure over time.

In summary, OA and SA may form excellent nutritional supplements to the diets of type 2 diabetics. The synergy of the two organic acids has been shown to improve glycaemic control in fructose-fed STZ-induced diabetic S-D rats through improving insulin sensitivity and a possible increase in insulin secretion from the pancreatic *β*-cells. The improved insulin sensitivity mitigated the suppression of insulin-dependent glucagon secretion and contributed to a decrease in the hepatic glucose output. Additionally, the cocktail highlighted alleviation of polydipsia and polyphagia. Furthermore, consumption of the cocktail yielded no change in normoglycaemic animals and may therefore be safe for consumption by nondiabetics, unlike many of the currently available antidiabetic drugs.

## 5. Conclusion

A type 2 diabetic rat model was successfully created as shown with the anti-diabetic drug response test (glibenclamide at 5 mg/kg BW) and the exhibition of insulin resistance. The two organic acids (OA and SA) synergistically improved symptoms associated with type 2 diabetes in STZ-induced diabetic rats. A reduction in nonfasting BGL was associated with improved insulin sensitivity whilst reducing polyphagia and polydipsia. However, the cocktail had no significant effect on diabetes-induced hypertension but its antidiabetic effect rivals that of a known antidiabetic agent, glibenclamide (10 mg/kg BW). With additional research, the cocktail may be introduced as a nutritional supplement that can be incorporated into the diets of diabetics to assist with mitigating the progressive nature of the disease.

## Figures and Tables

**Table 1:** Variation in serum insulin, glucagon, and insulin resistance in treated and untreated diabetic and nondiabetic Sprague Dawley rats.

	NC	NT	DC	DGLIB	DT
Serum insulin levels (ng/mL)	0.49 ± 0.06^a^	0.44 ± 0.06^a^	0.39 ± 0.05^a^	0.53 ± 0.05^a^	0.46 ± 0.05^a^
Serum glucagon levels (pg/mL)	17.29 ± 4.80^a^	26.66 ± 5.09^a^	55.50 ± 10.93^b^	40.12 ± 12.55^ab^	25.16 ± 6.82^a^
G/I × 10^3^	1.638 ± 0.246^a^	1.831 ± 0.197^a^	3.728 ± 0.556^b^	1.288 ± 0.138^a^	1.951 ± 0.263^a^
QUICKI	0.336 ± 0.006^a^	0.344 ± 0.002^a^	0.313 ± 0.004^b^	0.334 ± 0.004^a^	0.334 ± 0.002^a^

Data are expressed as mean ± standard error of the mean and analysed by Tukey post hoc test. NC: normal control, NT: normal treatment, DC: diabetic control, DT: diabetic treatment, and DGlib: diabetic glibenclamide group, where the letters represent the following: a, statistically significant differences when compared with the values in the same row with superscripts b; b, statistically significant differences when compared with the values in the same row with superscripts a. Significant difference is considered at *p* ≤ 0.05.

## Data Availability

Data will be made available upon reasonable request.

## References

[1] Bennett N. R., Francis D. K., Ferguson T. S. (2015). Disparities in diabetes mellitus among caribbean populations: a scoping review. *International Journal for Equity in Health*.

[2] American Diabetes Association (ADA) (2019). Classification and diagnoses of diabetes. *Diabetes Care*.

[3] World Health Organization (1999). Definition, diagnosis and diabetes mellitus and its complications: a report of a WHO consultation: part 1. *Diagnosis and Classification of Diabetes*.

[4] American Diabetes Association (ADA) (2004). Classification and diagnosis of diabetes mellitus. *Diabetes Care*.

[5] Idm’hand E., Msanda F., Cherifi K. (2020). Medical uses, phytochemistry and pharmacology of *Ammodaucusleucotrichus*. *Clinical Phytoscience*.

[6] Leon B. M., Maddox T. M. (2015). Diabetes and cardiovascular diseases: epidemiology, biological mechanisms, treatment recommendations and future research. *World Journal of Diabetes*.

[7] Kooti W., Farokhipour M., Asadzadeh Z., Ashtary-Larky D., Asadi-Samani M. (2016). The role of medicinal plants in diabetes: a systematic review. *Electronic Physician*.

[8] Goldin A., Beckman J. A., Schmidt A. M., Creager M. A. (2006). Advanced glycation end products. *Circulation*.

[9] International Federation of Diabetes North America and the Caribbean (2014). Prevalence of diabetes mellitus. https://www.idf.org/about-diabetes.

[10] Michie C. A. (1992). The use of herbal remedies in Jamaica. *Annals of Tropical Paediatrics*.

[11] Mitchell S. A. (2011). The Jamaican root tonics: a botanical reference. *Focus on Alternative and Complementary Therapies*.

[12] Picking D., Delgoda R., Vanderbroek I., Katerere D., Applequist W., Aboyade O. M., Togo C. (2019). Traditional knowledge systems ant the role of traditional medicine in Jamaica. *Traditional and Indigenous Knowledge for the Modern Era: A Natural and Science Perspective*.

[13] Fabricant D. S., Farnsworth N. R. (2001). The value of plants used in traditional medicine for drug discovery. *Environmental Health Perspectives*.

[14] World Health Organization [WHO] (2013). *Traditional Medicine*.

[15] Sasidharan S., Chen Y., Saravanan D., Sundram K. M., Latha L. Y. (2011). Extraction, isolation and characterization of bioactive compounds from plants’ extracts. *African Journal of Traditional, Complementary and Alternative Medicines*.

[16] Wang J., Zhang X., Lan H., Wang W. (2019). Effect of garlic supplement in the management of type 2 diabetes mellitus (T2DM): a meta-analysis of randomized controlled trials. *Food & Nutrition Research*.

[17] Alexander-Lindo R. L., Porter R. B. R., Nwokocha C. R., Lattibeaudiere K. G. (2020). The phytochemical and pharmacological screening of three crude extracts of desmodium canum (strong back). *Clinical Phytoscience*.

[18] Lattibeaudiere K., Porter R., Alexander-Lindo R. L. (2020). The isolation of hypoglycaemic compounds from desmodiumcanumand their synergistic effect on blood glucose levels in normal sprague-dawley rats. *Evidence-Based Complementary and Alternative Medicine*.

[19] Saravanan R., Pari L. (2007). Succinic acid monoethyl ester, a novel insulinotropic agent: effect on lipid composition and lipid peroxidation in streptozotocin-nicotin-amide induced type 2 diabetic rats. *Molecular and Cellular Biochemistry*.

[20] De Marchi U., Hermant A., Thevenet J. (2017). A novel ATP-synthase-independent mechanism coupling mitochondrial activation to exocytosis in insulin-secreting cells. *Journal of Cell Science*.

[21] Wilson R. D., Islam M. S. (2012). Fructose-fed streptozotocin-injected rat: an alternative model for type 2 diabetes. *Pharmacological Reports*.

[22] Pandarekandy S. T., Sreejesh P. G., Harikuman Thampi B. S., Sreekumaran E. (2017). Hypoglycaemic effect of glibenclamide: a critical study on the basis of creatinine and lipid peroxidation status of streptozotocin-induced diabetic rats. *Indian Journal of Pharmaceutical Sciences*.

[23] Cacho J., Sevillano J., de Castro J., Herrera E., Ramos M. P. (2008). Validation of simple indexes to assess insulin sensitivity during pregnancy in wistar and sprague-dawley rats. *American Journal of Physiology—Endocrinology and Metabolism*.

[24] Logan K., Asemota H., Nwokocha C. (2020). The effects of synthesized semicarbazone copper complex on blood pressure in normotensive and L-NAME induced hypertensive rats. *Journal of Biotechnology and Biomedicine*.

[25] Rutledge A. C., Adeli K. (2007). Fructose and the metabolic syndrome: pathophysiology and molecular mechanisms. *Nutrition Reviews*.

[26] Tran L. T., Yuen V. G., McNeill J. H. (2009). The fructose-fed rat: a review on the mechanisms of fructose-induced insulin resistance and hypertension. *Molecular and Cellular Biochemistry*.

[27] Glibenclamide B. L. F.. (2017). *Reference Module in Biomedical Sciences*.

[28] O’Meara N. M., Shapiro E. T., Van Cauter E., Polonsky K. S. (1990). Effect of glyburide on beta cell responsiveness to glucose in non-insulin-dependent diabetes mellitus. *Americas Journal of Medicine*.

[29] Foster S. R., Omoruyi F. O., Bustamante J., Lindo R. L. A., Dilworth L. L. (2016). The effect of combined inositol hexakisphosphate and inositol supplement in streptozotocin-induced type 2 diabetic rats. *International Journal of Experimental Pathology*.

[30] Rehman K., Haider K., Jabeen K., Akash M. S. H. (2020). Current perspectives of oleic acid: regulation of molecular pathways in mitochondrial and endothelial functioning against insulin resistance and diabetes. *Reviews in Endocrine & Metabolic Disorders*.

[31] Tomita T., Hosoda K., Fujikura J., Inagaki N., Nakao K. (2014). The protein- couples long chain acid receptor GPR40 and glucose metabolism. *Frontiers in Endocrinology*.

[32] Liu X., Kris-Etherton P. M., West S. G. (2016). Effects of canola and high-oleic-acid canola oils on abdominal fat mass in individuals with central obesity. *Obesity*.

[33] Khoo N. K. H., Fazzari M., Chartoumpekis D. V. (2019). Electrophilic nitro-oleic acid reverses obesity-induced hepatic steatosis. *Redox Biology*.

[34] Tutunchi H., Ostadrahimi A., Saghafi-Asl M. (2020). The effects of diets enriched in monounsaturated oleic acid on the management and prevention of obesity: a systematic review of human intervention studies. *Advances in Nutrition*.

[35] Colussi G., Da Porto A., Cavarape A. (2020). Hypertension and type 2 diabetes: lights and shadows about causality. *Journal of Human Hypertension*.

[36] Ferrannine E., Cushman W. C. (2012). Diabetes and hypertension: the bad companions. *Lancet*.

